# A study on patterns of use of mobile phone and nomophobia in medical undergraduate students during a COVID-19 pandemic lockdown

**DOI:** 10.1192/j.eurpsy.2021.1781

**Published:** 2021-08-13

**Authors:** P. Bhatnagar, S. Tarachandra, S. Undaru

**Affiliations:** Psychiatry, K.S. Hegde Medical Academy, Mangalore, India

**Keywords:** nomophobia, COVID-19 lockdown, Medical Students, mobile phone

## Abstract

**Introduction:**

The governments of various countries enforced a lockdown to contain the COVID -19 pandemic. As the colleges remain closed, the academic teachings for students was conducted online. The mobile phone remained the main source for academics and entertainment during this period.

**Objectives:**

To assess patterns of use of mobile phone by Medical Undergraduate students prior to and during the COVID-19 lockdown. To assess Nomophobia among same participants.

**Methods:**

This study was done by an online survey method after obtaining approval from the Institutional Ethics Committee. A validated questionnaire on patterns of mobile phone use and the Nomophobia Questionnaire(NMP-Q) was completed by the medical students (n=187) who consented to participate in the study

**Results:**

Prior to the pandemic lockdown, 52.9% of the participants used the mobile phones for 2-4 hours per day with 78% of the usage in social media. During lockdown, 89.3% of the participants reported an increase in the usage of mobile phones. 35.65% reported an increase in use by 2-4 hours everyday. About 30.5 % used the mobile phone for 6-8 hours per day. 80.2 % reported a maximum usage for social media. 59.45% reported a maximum usage for online academics. 33.7% frequently checked their phones once in 15 minutes. About 60.43% of the participants were in the moderate and 21.4% in the severe category of nomophobia.

**Conclusions:**

There is an increase in mobile phone usage during the lockdown with a significant proportion of students in the moderate and severe category of nomophobia.

**Disclosure:**

No significant relationships.

